# Taft's Operative Dentistry, Third Edition

**Published:** 1877-05

**Authors:** 


### BIBLIOGRAPHICAL.

*Taft s Operative Dentistry.*—Third Edition. Publishers: Lindsay & Blackiston, Philadelphia, Pa.

With pleasure we welcome a new edition of this valuable work, which, as the Author remarks in the preface, has involved little less labor than the writing of an entirely new book, so great have been the changes in almost every branch of practice during the past eight years. In the consideration of " Deposits," the origin of Tartar is treated of at greater length than in the former editions, and changes in the phraseology made in other sentences besides brief additions. The old illustration of scalers has been changed for another cut representing instruments better adapted for the purpose. The section on "Irregularity" remains the same, as also those relating to Atrophy, Exostosis, Abrasion, Denuding and Necrosis, Caries of the Teeth. The numbers of gold foil in most common use have been changed, 3, 5, 8, and 15 being omitted, and 60, and 240, added to the list; and among the materials used for filling teeth, oxy-chloride of zinc receives a separate notice. To the article on " Instruments for Filling," a number of new cuts of modern instruments are added, among them, those of the dental engine and its appliances, while the old drill stocks are omitted. In the article on " Separation of the Teeth," corundum disks, carriers and shields receive due attention, as well as " Jarvi's separators." Considerable matter is added to the chapter on " Filling Teeth," the cuts of " magnifiers" and " reflectors" being new, while the notice and illustration of " Forbes Gouges" are omitted as obselete. " Exclusion of Moistnre" takes the place of the more homely expression "drying cavities," this section being rewritten, and due attention is given to saliva extractors and rubber dam punches, clamps, clamp-forceps, etc., and the application of the rubber dam. The term " cohesive" is substituted for " adhesive" when speaking of gold foil. Some addition is made to the section on the " Mallet ;" also to that describing the finishing of fillings, relating chiefly to the use of Hindustan and Scotch stones, corundum cones, disks, etc.

Some omissions and additions are also found in the section relating to "filling by classes and modifications," and several old illustrations of cheek distenders and tongue depressors are left out.

Considerable additions are also found in the section on "filling by classes and modifications," and the matrices of Dr. Jack, are illustrated and their application described: also Osmond's screws for anchoring fillings, with illustrations. At end of this chapter, a second and improved method of inserting pieces of enamel in the labial surfaces of superior incisors, etc., is described, and reference made to the combination of gold and platinum for filling prominent cavities. In the chapter on "Sensitive Dentine," reference is made to the use of 'carvacrol' "dental pain obtunder," and the word "terchloride" properly rendered. Considerable is added to the chapter on "Exposed Pulps," and the latest methods of treating the different pathological conditions of the dental pulp, are described in full; with an illustration of Palmer's "nerve canal instruments". The section on "Alveolar Abscess" has also been rewritten and contains a great deal of valuable information. The chapter on "Pivot Teeth" remains the same. Wattling's forceps for the removal of left inferior molars, is the addition to the chapter on "extraction of teeth."

To the chapter on "Anæsthetics" is added a cut of a large gas holder for liquid nitrous oxide gas, and a description given of Von Bonhorst's dental pain obtunder and its "applicator" illustrated

To this edition an appendix is added relating to "Dental Caries," "Palmer's Plugging Instruments," "Mallets," with illustrations of Bonwill's Electro-magnetic Mallet, Buckingham's Automatic Plugger for engines, and Jack's Matrices, with directions for their use; and entering upon dental therapeutics, the last section of the appendix is devoted to the reprint of an article on Salicylic Acid, taken from the *Chemist and Druggist*. This additional matter and the new illustrations, greatly enhance the value of this third edition over the preceding ones, and will cause it to be gladly welcomed by the profession.

The style in which the work is gotten up by the Publishers, renders it very attractive, and the large clear type and good paper will be appreciated by the student.

---

